# Properties of Persian Translated Version of Body Compassion Scale

**DOI:** 10.1002/brb3.70845

**Published:** 2025-10-21

**Authors:** Nahid Hoseininezhad, Roya Rasuli, Abbas Abdollahi, Kavoos Hassanli

**Affiliations:** ^1^ Department of Counseling, Faculty of Education and Psychology Alzahra University Tehran Iran; ^2^ Department of Persian Language and Literature, Faculty of Literature and Humanities Shiraz University Shiraz Iran

**Keywords:** body compassion, body image, psychometrics, self‐compassion

## Abstract

**Purpose:**

The Body Compassion Scale (BCS) is a self‐report instrument developed to evaluate body compassion across three dimensions: defusion, common humanity, and acceptance. This study aimed to translate the BCS into Persian and assess its psychometric properties within an Iranian sample.

**Methods:**

A total of 650 participants (522 women and 128 men), aged 18–40, were recruited through convenience sampling. The psychometric evaluation included assessments of face validity, content validity, and construct validity, along with reliability analysis using composite reliability and Cronbach's alpha. Confirmatory factor analysis (CFA) was conducted to examine the factor structure and model fit of the BCS.

**Findings:**

The Persian version of the BCS demonstrated strong validity and reliability, with composite reliability and Cronbach's alpha values exceeding 0.7. CFA confirmed the three‐factor structure, with acceptable goodness‐of‐fit indices. Significant correlations between the BCS and related measures—including the Self‐Compassion Short Form, the Santa Clara Brief Compassion Scale, and body image assessments—provided evidence supporting both convergent and divergent validity.

**Conclusion:**

These results indicate that the Persian BCS is a valid and reliable tool for assessing body compassion among the Iranian populations.

## Graphical abstract

The Persian version of the Body Compassion Scale (BCS) demonstrated strong reliability and validity in the Iranian context. The scale evaluates body compassion across three dimensions—self‐kindness, mindfulness, and common humanity—and showed significant correlations with related measures. Findings highlight the role of body compassion in promoting emotional health and self‐compassion in Iranian culture.

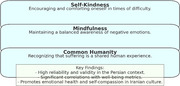

## 1 Introduction

In today's world, the body, face, and their appearance are important aspects of human identity. Over the past decades, research on body image has grown significantly (Stagi et al. 2021). People who are seen as more attractive are often judged to be kinder, more intelligent, and physically appealing. This perception can increase their chances of success in jobs, marriage, happiness, and overall life satisfaction (Stein et al. 2021). Body image is a broad and evolving concept. It encompasses an individual's perception of their own body size and physical fitness. It concerns how other people perceive and feel about that individual's body (Blashill and Wilhelm 2014). Body image includes a person's thoughts, feelings, and perceptions about their body. This covers how they look, how they feel about their body, and how they think others perceive them (Grogan 2006). A person may overestimate or underestimate their body size. This misperception can cause emotional and cognitive changes, leading to dissatisfaction and worry about their appearance. Body image is a psychological concept. It plays an important role for health psychologists (Alleva and Tylka 2021). One key part of body image is dissatisfaction. This occurs when people negatively evaluate their body's size, shape, or weight. It reflects the difference between their real body and their ideal body image (Fardouly and Vartanian 2016). Worry about body image is a serious problem. It is linked to mental health issues like low self‐esteem, depression, and anxiety (Şanlier et al. 2016). Having a negative view of oneself can cause body dissatisfaction and feelings of unattractiveness. In some cases, it can lead to an obsession with appearance that interferes with normal functioning (Richetin et al. 2012).

In recent years, the realm of body image has attracted significant interest from researchers. Various tools have been devised to measure body image, including the Multidimensional Body‐Self Relations Questionnaire (MBSRQ). The 46‐item questionnaire was designed by Cash et al. in 1986 and 1987 to evaluate body image (Cash et al. 1997). Another notable scale is a 19‐question survey about body image that addresses an individual's dissatisfaction and concern about their appearance, devised by Littleton et al. (2005), which has demonstrated good reliability and validity. The Body Image Guilt and Shame Scale was developed by Thompson et al. (2003) and consists of 15 questions that assess individuals' tendencies toward shame and guilt about their body image through scenarios regarding weight and body. Also, the Body Appreciation Scale, developed by Avalos et al. (2005) investigates positive attitudes toward the body. Although all these tools measure body image, they overlook the role of self‐compassion regarding body image. Studies have indicated a significant relationship between self‐compassion and body image (Murn and Steele 2020; Pullmer et al. 2019). Self‐compassion is an adaptive strategy for regulating emotions. It involves being sensitive and willing to reduce one's own pain and suffering. It also includes the ability to treat oneself with kindness and understanding when facing difficulties, setbacks, or personal failures. This approach contrasts with engaging in harsh self‐criticism (Neff and Dahm 2015). Compassion entails adopting a mindful attitude, instead of excessively recognizing and fixating on failures and personal limitations. Moreover, compassion calls for a sense of common humanity, opposing isolation, and entails recognizing that all human beings have limitations, are imperfect, and are prone to setbacks or mistakes in life (Yarnell and Neff 2013).

The concept of body compassion was developed to combine elements of body image and self‐compassion. This idea is founded on international studies related to body image. It includes three main parts of self‐compassion: (1) self‐kindness, which means treating oneself with kindness and understanding instead of harsh self‐criticism; (2) common humanity, which involves recognizing that one's experiences are shared by others, thus promoting connection; and (3) mindfulness, which is about keeping a balanced awareness of negative thoughts and feelings without becoming overwhelmed (Neff 2003; Nooripour et al. 2022). At the same time, the idea of the “self” has shifted from a general concept to specifically include the “physical self” (Altman et al. 2020). Understanding and measuring this concept helps provide insights into behaviors that improve mental health, well‐being, and quality of life. The Body Compassion Scale (BCS) is an effective tool for measuring this concept and its three parts (Altman et al. 2020).

So far, only a few studies have examined whether the factor structure of the BCS is valid. However, two relevant studies have been found that address this topic. One of these studies took place in Italy. It evaluated the validity and reliability of an Italian version of the BCS. The study included 695 Italian women as participants (Policardo et al. 2022). Using confirmatory factor analysis (CFA), the study confirmed that the BCS retained its original three‐factor structure. This structure was initially proposed by Altman et al. (2020). The results showed strong internal consistency within all three subscales of the BCS. However, the study found a weak relationship (covariance of 0.13) between the acceptance and common humanity subscales. It also found no significant relationship (covariance of −0.07) between the defusion and common humanity subscales. These findings differ from the original research, which showed moderate to strong and significant relationships among all subscales. Another study was conducted with Australian women. In this study, 513 female participants supported the validity of the BCS's three‐factor structure as well (Van Niekerk et al. 2023). This study also found that the defusion and acceptance subscales had stronger links to body image measures than to self‐compassion measures. Despite these studies, there is currently no established standard or tool to assess body compassion among Persian‐speaking populations.

Although previous studies have confirmed the validity of the BCS among Italian and Australian women (Policardo et al. 2022), no research has yet examined its psychometric properties in Persian‐speaking Iranian populations. Cultural norms, values, and language nuances significantly influence how concepts like body compassion are understood and expressed (Chang et al. 2021; Heinke and Louis 2009; Payne 2013). Therefore, it is crucial to assess the reliability and validity of the scale within the specific cultural context of this population. Iran has a unique social and cultural environment that encompasses prevalent attitudes toward body image, gender roles, and self‐compassion. These factors may affect how participants respond to the scale and could influence the factor structure of the BCS. Applying the scale without proper local validation may produce inaccurate results. Such inaccuracies can lead to misleading conclusions in both research and clinical practice. Lacking a validated Persian version prevents Iranian researchers and clinicians from accurately measuring body compassion. This limitation slows the creation of culturally suitable interventions that promote body‐related health and well‐being. Therefore, this study aims to address this important gap by translating, adapting culturally, and psychometrically testing the BCS in a sample from Iran. This work will offer a reliable instrument for future research and help improve understanding and encouragement of body compassion among Persian‐speaking individuals. This study seeks to address this critical gap by translating, culturally adapting, and psychometrically validating the BCS in an Iranian sample. This effort will provide a reliable and culturally sensitive instrument for use in research and clinical settings, ultimately supporting body‐related mental health and well‐being in Persian‐speaking populations.

The present study aims to evaluate the construct validity, internal consistency, and factorial structure of the Persian‐translated version of the BCS in an Iranian population.

## 2 Methods

The present research employed a descriptive correlational methodology.

### 2.1 Participants

This study involved 650 Iranian university students who participated voluntarily and were recruited using convenience sampling between June 21, 2023, and February 25, 2024. The statistical population included individuals aged 18 and older, of both genders. The sample initially consisted of 688 respondents (552 women and 136 men), from which 650 valid responses (522 women and 128 men) were retained after data screening. Participants were required to have adequate reading and writing skills, provide informed consent, and have no history of chronic physical or psychological disorders. Those who reported current use of psychiatric medication or long‐term illness were excluded from the study. All data were self‐reported. Participants were recruited through a range of online communication platforms such as WhatsApp and Telegram, reflecting the practical accessibility of the student population. Participants were invited via messages and links shared in university‐related online groups and social networks popular among students. The gender imbalance, with a higher proportion of female participants, was noted as a limitation arising from the non‐random sampling method.

### 2.2 Procedure

The BCS was translated into Persian using Brislin's (1980) back‐translation method (Brislin 1976; Jones et al. 2001). Two bilingual experts—one with a background in psychology and the other in English language studies—carried out the translation and back‐translation independently. The translator responsible for the back‐translation was blinded to the original English version. The preliminary Persian version underwent face validity review by two psychology experts and three university faculty members. Discrepancies were discussed and resolved to ensure conceptual and linguistic equivalence. To assess the clarity, cultural appropriateness, and comprehensibility of the translated items, a pilot test was conducted with 25 participants selected via convenience sampling. Feedback from the pilot group was used to refine item wording, clarify ambiguous terms, and enhance the overall clarity of the scale. Participants were informed about the confidentiality of their responses, and completion of the questionnaire was entirely voluntary. The final questionnaire was administered online between June 2023 and February 2024 using Google Forms. Links were shared via widely used messaging platforms in Iran (WhatsApp and Telegram). All items were mandatory to prevent incomplete responses. Participants could review and modify their responses before submission. They were given 14 days to complete the form, with reminders sent periodically to maximize the response rate.

Responses were screened for missing data, inconsistencies, and outliers using SPSS‐26. Invalid responses were removed. No personally identifiable information was collected, and all data were securely stored in password‐protected, encrypted files accessible only to the research team. Ethical approval was obtained from Alzahra University's Institutional Review Board. Following data cleaning, psychometric analyses were conducted to evaluate the scale's reliability and validity.

### 2.3 Questionnaires

#### 2.3.1 Body Compassion Scale (BCS)

The BCS was developed by Altman et al. (2020). It consists of 23 items, organized into three subscales: defusion, common humanity, and acceptance. An example item from the defusion subscale is “When I get disappointed and I feel frustrated with my body's inability to do something, I tend to feel separate and cut off from other people.” Items from the common humanity subscale include “When I am frustrated with some aspect of my appearance, I try to remind myself most people feel this way at some time.” An example item from the acceptance subscale is “I am accepting my looks just the way they are.” It employs a Likert point scale ranging from 1 (*almost never*) to 5 (*almost always*). The scale has been developed for individuals aged 15 years and older who possess the ability to read and write. The developers of questionnaire demonstrated high internal consistency with Cronbach's alpha coefficients of 0.92, 0.91, and 0.87 for the defusion, common humanity, and acceptance subscales, respectively. These results highlight the strong validity and reliability of this measurement tool.

#### 2.3.2 Self‐Compassion Scale‐Short Form (SCS‐SF)

This scale was developed by Raes et al. (2011) and consists of 12 items rated on a five‐point Likert scale (0 = *almost never* to 5 = *almost always*). Higher scores indicate higher levels of self‐compassion. The scale measures three binary components across six subscales: self‐kindness (reverse‐coded self‐critical), mindfulness (reverse‐coded over‐identification), and common humanity versus isolation. Example items include “When something painful happens, I try to have a balanced view of the situation,” assessing respondents' reactions to difficult circumstances. Scores on certain items are reverse coded, specifically 12, 11, 9, 8, 4, and 1. The short form of the Self‐Compassion Scale (SCS) shows a strong correlation (*r* ≥ 0.97) with the original version, supporting its reliability and validity. This high correlation is further bolstered by a retest reliability of 0.92. In the present study, the scale demonstrated good internal consistency with a Cronbach's alpha coefficient of 0.79, suggesting reliable measurement.

#### 2.3.3 Santa Clara Brief Compassion Scale (SCBCS)

This scale is a short version of the Compassionate Love Scale (Sprecher & Fehr 2005) and comprises five items to assess compassion and its association with social behaviors. It was developed by Hwang et al. (2008). This scale includes statements like “When I hear about someone (a stranger) going through a difficult time, I feel a great deal of compassion for him or her,” rated on a seven‐point Likert scale (0 = *not at all true* to 7 = *very true*). Scores on the scale range from 5 to 35. The original version of the SCBCS demonstrated high internal consistency with a Cronbach's alpha coefficient reported as 0.90. In the present study, the scale exhibited a Cronbach's alpha reliability coefficient of 0.87, indicating strong internal consistency.

#### 2.3.4 Body Image‐Acceptance and Action Questionnaire (BIAAQ)

The scale was initially developed by Sandoz et al. in the year 2013 and has since undergone several modifications and adaptations, resulting in its current existence in diverse formats and versions. The present study employed 12‐item version. Items such as “worrying about my weight makes it difficult for me to live a life that I value” are rated on a seven‐point Likert scale (0 = *never true* to 7 = *always true*), resulting in scores ranging from 12 to 84. Higher scores indicate greater inflexibility in body image (Sandoz et al. 2013). In this study, the BIAAQ demonstrated good reliability with a Cronbach's alpha coefficient of 0.87, indicating strong internal consistency. We also assessed the scale's face and content validity using Cronbach's alpha in a retest scenario, yielding coefficients of 0.93 and 0.90.

### 2.4 Statistical Analysis

Statistical analysis was conducted using SPSS software version 24 and R version 4.3.3. Descriptive analyses were performed using SPSS, while R version 4.3.3 (Packages: semPlot, lavaan, psych, semTools, and psycModel) was employed for conducting factor analysis, assessing composite reliability (CR), average variance extracted (AVE), and invariance testing.

## 3 Results

The questionnaires were completed by 522 women and 128 men, categorized by age brackets of 18–22 years (30.2%), 23–26 years (20.8%), 27–30 years (13.7%), and 31–40 years (35.4%) (Table 1).

**TABLE 1:** Demographic information about research sample.

Variable	Category	Abundance	Percentage (%)
**Age**	18–22	196	30.2
	23–26	135	20.8
	27–30	89	13.7
	31–40	230	35.4
	No answer	0	0
	**Total**	650	100
**Gender**	Female	522	80.3
	Male	128	19.7
	No answer	0	0
	**Total**	650	100
**Education**	BA/BS student	470	72.3
	MA/MS student	104	16.0
	PhD student	1	0.2
	BA/BS	4	0.6
	MA/MS	1	0.2
	PhD	18	2.8
	No answer	52	8.0
	**Total**	650	100

### 3.1 Face Validity

The qualitative and quantitative methods were used to assess the face validity of the BCS. The qualitative assessment involved a panel of five experts, consisting of two psychologists and three university professors. Their task was to evaluate the questionnaire for difficulty levels, clarity of wording, and semantic coherence. Expert feedback resulted in minor adjustments to certain questionnaire items. The quantitative face validity assessment involved rating each of the 23 questions on a five‐point Likert scale (ranging from 5 = *I fully agree* to 0 = *I fully disagree*). Subsequently, the questionnaire was administered to a pilot group of 10 individuals to further validate its clarity. Following completion by the pilot group, the impact score for each question was calculated to ascertain its face validity. The analysis confirmed that all questions exhibited satisfactory levels of face validity.

### 3.2 Content Validity

Content validity assesses the extent to which a research instrument's items comprehensively represent the target construct. Two primary methods exist for evaluating content validity: qualitative and quantitative approaches. The qualitative content validity evaluation involves soliciting expert feedback. In this study, five experts meticulously reviewed the scale items and provided written corrective feedback. Their assessment encompassed factors such as language grammar, vocabulary usage, question significance, item order, and completion time. Necessary revisions were implemented based on the expert feedback. The quantitative content validity evaluation ensures the selection of the most relevant and significant content. Two methods were employed in this study: the content validity ratio (CVR) and the content validity index (CVI). The CVR assessed the necessity of each item. Twelve experts analyzed the scale's transactional behavior, rating each item on a three‐point Likert scale (1 = *not necessary*; 2 = *useful but not necessary*; 3 = *necessary*). Lawshe's (1975) table was used to determine the significance level (*p* < 0.05) for item inclusion. Given the number of experts, an index value exceeding 62% indicated a necessary and significant item. All items achieved coefficients above 62%, and no elimination was necessary. The CVI assessed the clarity, relevance, and simplicity of each item. Experts evaluated the items on a four‐point Likert scale based on the criteria of “simplicity and smoothness,” “relevance,” and “clarity and transparency” (1 = *irrelevant*; 2 = *needs serious review*; 3 = *relevant but needs review*; 4 = *completely relevant*) (Almanasreh et al. 2019; Yusoff 2019). The item score above 0.79 is considered acceptable (Yusoff 2019). No items required modification or elimination based on the CVI scores (Table 2).

**TABLE 2:** Content validity ratio (CVR) and content validity index (CVI) for items of BCS.

No.	Item	Mean	SD	CVI—Clarity	CVI—Relevance	CVI—Simplicity	CVR—Necessity
**1**	When I feel frustrated with my body's inability to do something, I tend to feel separate and cut off from other people.	2.20	1.16	0.93	1.00	0.93	1.00
**2**	When I think about my body's inadequacies, it tends to make me feel more separate and cut off from other people.	2.06	1.15	1.00	1.00	1.00	1.00
**3**	When I think about my body's inadequacies, it tends to make me feel more separate and cut off from other people.	2.05	1.12	0.93	0.93	0.93	1.00
**4**	When my body fails at something important to me, I become consumed by feelings of inadequacy.	2.05	1.15	1.00	1.00	1.00	1.00
**5**	When my body is not responding the way I want it to, I tend to be tough on myself.	2.41	1.19	1.00	1.00	1.00	1.00
**6**	When I wish some aspect of my body looked different, it feels like no one else understands my struggle.	2.30	1.13	1.00	1.00	1.00	1.00
**7**	When I have physical symptoms, illness, or injury, it tends to make me feel more separate and cut off from other people.	1.90	1.08	1.00	1.00	1.00	1.00
**8**	When I notice aspects of my body that I do not like, I get down on myself.	2.20	1.21	0.93	0.93	0.93	1.00
**9**	When I am feeling physically uncomfortable, I tend to obsess and fixate on everything that is wrong.	2.02	1.11	1.00	0.93	1.00	1.00
**10**	When I am frustrated with some aspect of my appearance, I try to remind myself most people feel this way at some time.	2.85	1.20	0.93	0.93	0.93	1.00
**11**	When I doubt my ability to do a new physical activity, I try to remind myself that most people also feel this way at some point.	2.89	1.17	1.00	1.00	1.00	1.00
**12**	When I feel out of shape, I try to remind myself that most people feel this way at some point.	2.69	1.22	0.93	0.93	0.93	1.00
**13**	I try to see my body's failings as something everyone experiences in one way or another.	3.09	1.26	1.00	1.00	1.00	1.00
**14**	When I am injured, ill, or have physical symptoms, I remind myself that there are lots of other people in the world feeling like me.	3.33	1.21	1.00	1.00	1.00	1.00
**15**	When I feel frustrated with my body's inability to do something, I try to remind myself that most people in my condition feel this way at some point.	3.14	1.20	1.00	1.00	1.00	1.00
**16**	When I feel my body is inadequate in some way, I try to remind myself that feelings of inadequacy are shared by most people.	3.09	1.20	1.00	1.00	1.00	1.00
**17**	When I am at my lowest during times of physical symptoms, illness, or injury, I know I am not alone in feeling this way.	3.35	1.16	1.00	1.00	1.00	1.00
**18**	When I am concerned if people would consider me good‐looking, I remind myself that most everyone has the same concern.	2.91	1.20	1.00	1.00	1.00	1.00
**19**	I am accepting of my looks just the way they are.	4.06	1.07	1.00	1.00	1.00	1.00
**20**	I am accepting of the way I look without my clothes on.	3.76	1.23	1.00	1.00	1.00	1.00
**21**	I feel okay in my body.	3.85	1.16	0.93	1.00	0.93	1.00
**22**	I am tolerant of my body's flaws and inadequacies.	3.77	1.13	1.00	1.00	1.00	1.00
**23**	I am tolerant of the way my clothes fit me.	2.80	1.30	1.00	1.00	1.00	1.00

*Note*: CVI: Content validity index—*F*:ne/N; CVR: Content Validity; Ratio—*F*:(ne−N/2/N/2).

Examining the mean and standard deviation of the BCS's items indicates that the mean scores range from 1.90 to 4.6, with standard deviations ranging from 1.08 to 1.3. This study used skewness and kurtosis indices to assess the normality of the data. The analysis revealed that the skewness values ranged from −1.10 to 1.10, and the kurtosis values ranged from −1.21 to 0.70 (Table 3). These values, considering the acceptable thresholds for skewness (±2) and kurtosis (±5), indicate that the data distribution is normal. The frequency of response options (Table 3) demonstrates a clear pattern based on the response frequencies.

**TABLE 3:** Skewness, kurtosis, and frequency of response options for BCS's items.

Item	Skewness	Kurtosis	5 (Almost always)	4 (Always)	3 (Usually)	2 (Never)	1 (Almost never)
1	0.769	−0.44	26	96	77	233	218
2	0.977	−0.008	27	74	70	218	261
3	0.926	−0.10	20	77	77	215	261
4	0.976	−0.04	24	79	64	219	264
5	0.459	−0.93	26	134	94	225	171
6	0.921	−0.17	19	79	78	200	274
7	1.090	0.222	13	71	61	197	308
8	0.766	−0.577	29	108	60	222	231
9	0.906	−0.12	19	66	100	187	278
10	−0.04	−1.11	42	197	137	167	107
11	0.12	−1.12	34	219	131	171	95
12	0.114	−1.10	40	161	145	166	138
13	−0.20	−1.11	80	219	116	147	88
14	−0.47	−0.80	94	279	84	132	61
15	−0.31	−0.99	66	250	118	143	73
16	−0.22	−0.99	69	221	138	145	77
17	−0.50	−0.761	87	284	101	128	50
18	−0.08	−1.10	47	206	133	167	97
19	−1.07	0.248	277	233	52	75	13
20	−0.68	−0.68	235	189	93	99	34
21	−0.85	−0.28	229	239	65	92	25
22	−0.72	−0.43	198	247	86	96	23
23	0.205	−1.21	74	166	81	215	114

### 3.3 Construct Validity

Based on the three factors in the original study (Altman et al. 2020), CFA was conducted to examine the first‐order three‐factor solution. The three‐factor CFA was tested using R software version 4.3.3. The validity of a model can be assessed using criteria known as goodness‐of‐fit indices. The acceptable thresholds for these fit indices are as follows: *χ* ^2^ to degrees of freedom ratio (CMIN/df < 5), root mean square error of approximation (RMSEA < 0.08), standardized root mean square residual (SRMR < 0.1), Incremental Fit Index (IFI > 0.9), Tucker–Lewis Index (TLI > 0.9), and Comparative Fit Index (CFI > 0.9) (Byrne 2013). The goodness‐of‐fit results indicated a *χ* ^2^ value of 769.508, degrees of freedom (df) of 224, and a *χ* ^2^ to degrees of freedom ratio of 3.43. The RMSEA was 0.061 (with a 90% confidence interval (CI) of 0.057–0.066), and the SRMR was 0.069, indicating a good model fit. The IFI for the three‐factor model was 0.944, the TLI was 0.936, and CFI was 0.944, all of which confirm the adequacy of the three‐factor model fit.

**FIGURE 1:**
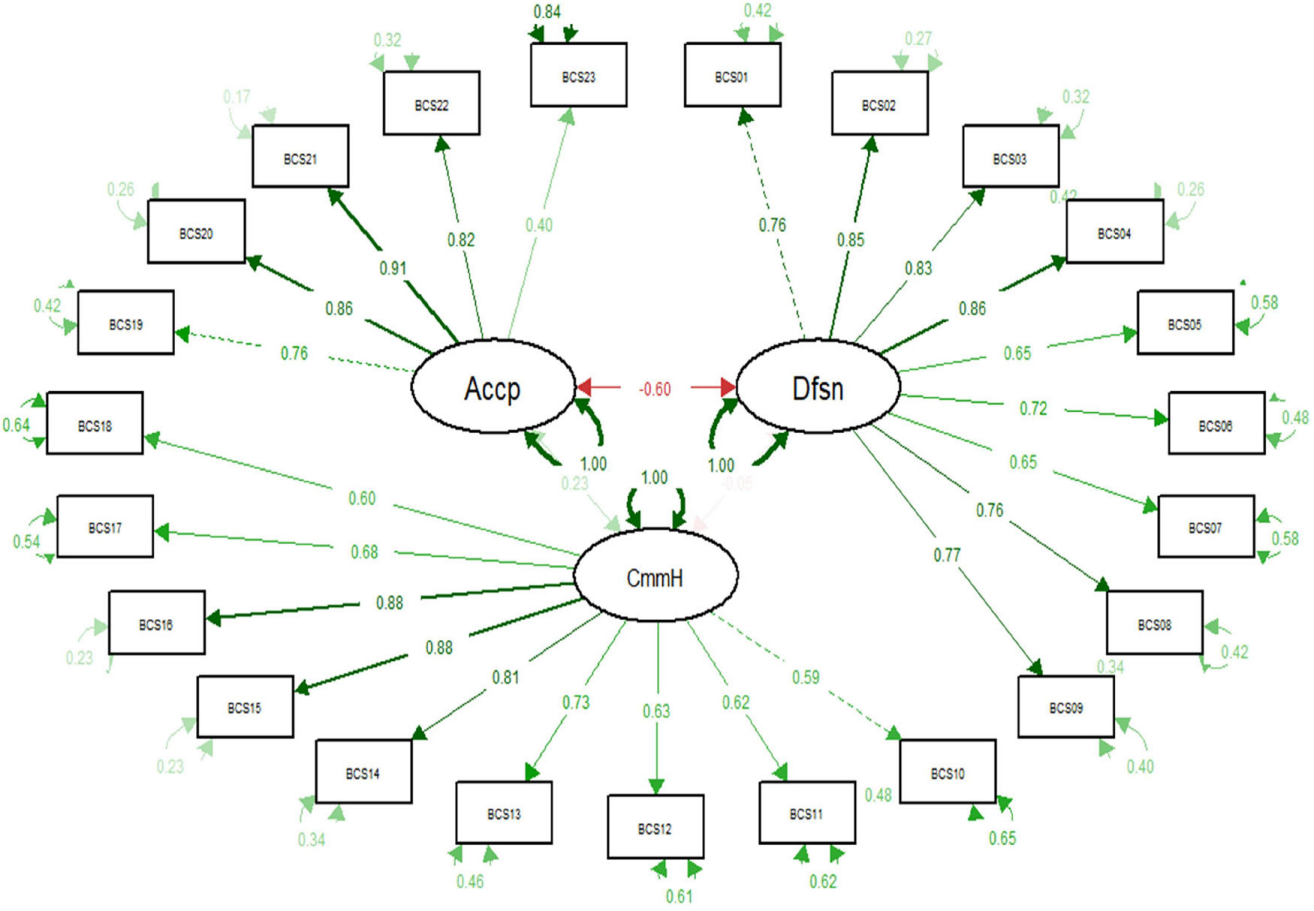
Confirmatory factor analysis (CFA) of Body Compassion Scale (BCS) and factor loadings.

The three‐factor CFA structure of the BCS is presented in Figure 1 and Table 4. The results indicated that all items of the BCS have very high factor loadings on their respective factors. These findings are statistically significant at the 95% confidence level, as indicated by the *Z* statistics and *p*‐values.

**TABLE 4:** Items, standardized loadings, Z scores, and significance levels of factor structure of BCS.

Factor	Item	Factor loading	*p*‐value	*Z*‐value
Defusion	1	0.761	—	—
	2	0.854	23.1	0.0001
	3	0.828	22.16	0.0001
	4	0.859	23.21	0.0001
	5	0.646	16.75	0.0001
	6	0.72	18.94	0.0001
	7	0.651	16.9	0.0001
	8	0.763	20.18	0.0001
	9	0.773	20.49	0.0001
Common humanity	10	0.594	—	—
	11	0.618	18.20	0.0001
	12	0.626	13.28	0.0001
	13	0.732	14.86	0.0001
	14	0.812	15.89	0.0001
	15	0.877	16.65	0.0001
	16	0.875	16.63	0.0001
	17	0.675	14.04	0.0001
	18	0.603	12.91	0.0001
Acceptance	19	0.759	—	—
	20	0.863	23.13	0.0001
	21	0.911	24.48	0.0001
	22	0.824	21.93	0.0001
	23	0.399	9.94	0.0001

In addition to assessing standardized factor loadings, many studies have used the criterion proposed by Fornell and Larcker (1981) to assess convergent validity. Fornell and Larcker (1981) suggested that convergent validity is established when a construct explains more than half of the variance in its related indicators. They proposed using the AVE to represent the average amount of variance that a construct explains in its indicators relative to the total variance in its indicators (Cheung et al. 2023).

The AVE method was used to assess the convergent validity of the three factors of the BCS, with AVE values greater than 0.50 indicating convergent validity of the scale factors. To evaluate reliability, Omega reliability, CR, and Cronbach's alpha were utilized. If the Omega reliability, CR, and Cronbach's alpha coefficients for a tool are all above 0.70, the tool is considered to have adequate reliability (Adriani et al. 2020). The analysis demonstrates that the BCS exhibits convergent validity and adequate reliability (Table 5).

**TABLE 5:** Extracted average variance, composite reliability, omega, and Cronbach's alpha for factors of BCS.

Variable	Cronbach's alpha	Composite reliability (CR)	McDonald's omega	Average variance extracted (AVE)
Defusion	0.92	0.90	0.928	0.60
Common humanity	0.90	0.87	0.908	0.55
Acceptance	0.85	0.81	0.867	0.52
Total BCS	0.80	0.95	—	0.54

*Note*: Composite reliability (CR) = $\frac{{\left(\sum \lambda_{i}\right)}^{2}}{{\left(\sum \lambda_{i}\right)}^{2}+\left(\sum \varepsilon_{2}\right)}$; average variance extracted (AVE)= $\frac{\sum \lambda_{I}^{2}}{n}$.

### 3.4 Concurrent Validity

The correlation matrix analysis of the study variables indicates that, as anticipated, the SCS exhibits a negative correlation with the BIAAQ. At the same time, it demonstrates a positive correlation with the Self‐Compassion Body Compassion Scale. The factors of the BCS show high correlations with the SCS and the BIAAQ, and a low correlation with the Self‐Compassion Body Compassion Scale. Specifically, SCS exhibits negative and positive correlations of −0.61 and 0.52 with defusion and acceptance. Similarly, BIAAQ demonstrates positive and negative correlations of 0.62 and −0.54 with defusion and acceptance (Table 6).

**TABLE 6:** Correlation between variables under study.

Variable	Mean	SD	Diffusion	Common humanity	Acceptance	SCS	SCBCS	BIAAQ
Diffusion	18.91	8.23	1					
Common Humanity	27.33	8.25	0.024	1				
Acceptance	18.23	4.72	−^**^0.53	^**^0.18	1			
SCS	40.56	7.58	−^**^0.61	^**^0.28	^**^0.52	1		
SCBCS	23.85	7.37	0.02	0.07	^*^0.09	.02	1	
BIAAQ	27.5	12.88	^**^0.62	*0.094	−^**^0.54	^**^−0.41	^*^0.08	1

**, * denote significant correlation at the 0.01 level (two‐tailed) and 0.05 level (two‐tailed), respectively.

Abbreviations: BIAAQ, Body Image Acceptance and Action Questionnaire; SCBCS, Santa Clara Brief Compassion Scale; SCS, Self‐Compassion Scale.

The variable of gender was measured to investigate the factor structure of the BCS within gender subgroups. Measurement invariance, a statistical technique for multigroup analysis, was employed. This technique involves three stages. The first stage is configural invariance, which establishes whether the same set of items and factors is identified for each group. The results of the configural invariance model in this study support the fit of the models, indicating equal factor structures across the two gender groups (*χ* ^2^/df = 2.94, CFI = 0.909, RMSEA = 0.077, SRMR = 0.071). The next stage is metric invariance, which examines whether the items of the scale have the same relationship with underlying factors across groups. The indices of metric invariance suggest that there is no difference in the relationship between the items and factors in the BCS across genders (*χ* ^2^/df = 2.85, CFI = 0.91, RMSEA = 0.076, SRMR = 0.073). Finally, scalar invariance was examined to assess the equality of intercepts and means across groups. The results support scalar invariance across groups (*χ* ^2^/df = 2.81, CFI = 0.908, RMSEA = 0.075, SRMR = 0.074). The fit indices of measurement invariance indicate that the factor structure of the BCS is equivalent and invariant across gender subgroups (Table 7).

**TABLE 7:** Measurement invariance for gender.

Analysis type	*Χ* ^2^	df	*p*‐value	CFI	RMSEA	SRMR	TLI
Configural	1338.19	454	0.0001	0.909	0.077	0.071	0.90
Metric	1354.93	474	0.0001	0.91	0.073	0.073	0.904
Scalar	1390.77	494	0.0001	0.908	0.074	0.074	0.906

## 4 Discussion

The current study investigated the psychometric properties of the Persian translation of the BCS in an Iranian sample. This research represents the first endeavor to assess the statistical properties of the BCS within the Iranian context, thereby offering valuable insights into the instrument's cross‐cultural applicability.

CFA was employed to evaluate the construct validity of the BCS subscales (defusion, common humanity, and acceptance). The findings supported the original three‐factor structure, with CR coefficients exceeding the recommended threshold of 0.70. Specifically, the defusion, common humanity, and acceptance subscales demonstrated CR values of 0.90, 0.87, and 0.81, respectively. These results provide evidence for the desirable construct validity of the Persian BCS. The internal consistency analysis using Cronbach's alpha coefficients revealed adequate reliability for all subscales.

Correlation analyses provided additional information regarding the scale's concurrent validity. The acceptance subscale exhibited a positive and statistically significant correlation with the Santa Clara SCS (*p* < 0.05). Conversely, the defusion and common humanity subscales did not demonstrate significant relationships with this measure (*p* < 0.01). Interestingly, the defusion subscale displayed a negative correlation with the SCS (*p* < 0.01), while the acceptance and common humanity subscales showed positive and statistically significant relationships (*p* < 0.01). These findings align with theoretical expectations, suggesting that acceptance and common humanity constitute core elements of self‐compassion, whereas defusion may represent a distinct coping mechanism.

In comparison, Policardo et al. (2022) reported weak or nonsignificant relationships between defusion and other subscales, indicating that defusion may not integrate as strongly into the broader construct of self‐compassion. However, our finding of a negative correlation between defusion and acceptance diverges from their data, suggesting that cultural context may influence how individuals relate to these constructs.

In the present study, however, the negative correlation between defusion and acceptance was statistically significant (*p* < 0.01), potentially indicating that cultural differences may shape how these constructs are related. These discrepancies may stem from divergent cultural values regarding self‐perception and bodily awareness in Iranian versus Western populations.

Moreover, the defusion subscale exhibited a positive correlation with body image flexibility, as measured by the BI‐AAQ. However, the observed relationship between defusion and acceptance was negative, potentially indicating that these constructs reflect contrasting approaches to body image. The common humanity subscale also demonstrated a positive association with body image flexibility (*p* < 0.05).

This pattern is consistent with findings from Van Niekerk et al. (2023), who reported that the defusion and acceptance subscales of the BCS were more strongly associated with body image variables than with traditional self‐compassion measures among Australian females (Van Niekerk et al. 2023). Such consistency suggests that defusion may serve a more prominent role in body‐related psychological flexibility, particularly in cultural contexts where bodily concerns are closely tied to self‐worth.

One possible explanation for variations in subscale relationships across studies lies in the demographic composition of samples. Policardo et al. (2022) and Van Niekerk et al. (2023) exclusively examined female participants, whereas the current study included both men and women, which may influence overall patterns of body‐related self‐compassion. Furthermore, Iran's sociocultural emphasis on modesty, collectivism, and gendered expectations around appearance may affect how participants interpret items related to self‐kindness, common humanity, and defusion. These contextual nuances highlight the need for further comparative research to better understand how body compassion manifests across cultures. The relationships are consistent with prior research. Acceptance, encompassing self‐recognition as a human being with inherent flaws, is associated with self‐acceptance and reduced self‐criticism, fostering greater self‐compassion during challenging situations (Neff and Dahm 2015).

The self‐compassion, characterized by the recognition of shared human limitations and imperfections, promotes self‐acceptance (Yarnell and Neff 2013). Conversely, defusion, which can serve as a coping strategy, might lead individuals to perceive themselves as distinct from others, potentially increasing self‐criticism and diminishing self‐compassion (Murn and Steele 2020). Likewise, body image flexibility facilitates self‐acceptance by promoting the avoidance of self‐isolation and fostering the acceptance of oneself in totality. Together, these findings suggest that while the BCS maintains its core psychometric strengths across contexts, the relative contribution of its subscales may be modulated by cultural factors, gender norms, and population characteristics.

Together, these culturally nuanced findings underscore the importance of continued comparative validation across different sociocultural contexts to fully understand the multidimensional nature of body compassion.

Overall, the findings of this study are consistent with the work of Altman et al. (2020), who proposed the BCS as a valid instrument for measuring body compassion. This research provides preliminary evidence for the desirable validity and reliability of the Persian version of the BCS, particularly within the Iranian student population. These findings suggest that the BCS can be confidently employed to assess body compassion in Iranian samples, enabling the evaluation and development of appropriate preventive interventions.

By explicitly comparing the current results with international findings from Italy and Australia, the present study enriches the global understanding of the BCS's structural validity and cultural adaptability. The BCS serves as a valuable tool for further research in Iran, as the integration of body image and compassion components may positively contribute to self‐acceptance. By comparing the current findings with existing international validations, this study enhances the global understanding of how body compassion operates across cultures and further establishes the BCS as a reliable instrument adaptable to diverse populations. By measuring body compassion, researchers can gain valuable insights and pave the way for the development of necessary interventions and programs.

As with any research endeavor, the present study has several limitations that warrant consideration. The reliance on Cronbach's alpha coefficient for assessing reliability, although common, represents a single measure of internal consistency and does not evaluate test‐retest reliability over time. Future research should consider employing additional reliability measures to strengthen the robustness of findings. The study sample predominantly consisted of university students, which may limit the generalizability of results to broader segments of the Iranian population. Therefore, subsequent studies should strive to include more diverse participant demographics. This would enable a more comprehensive understanding of how body compassion manifests across different age groups, socioeconomic statuses, and cultural contexts within Iran. Despite efforts to ensure translation accuracy and face validity of the BCS, the present study did not conduct formal re‐testing to assess the stability and consistency of measurements over time. Addressing this limitation in future investigations would enhance the applicability and reliability of the BCS among diverse Iranian populations.

Future research should also explore several critical areas that can deepen our understanding and application of the BCS in Iran. Longitudinal studies are essential to examine the stability of body compassion over time and its long‐term implications for mental health. Such studies could clarify whether interventions aimed at enhancing body compassion produce sustained benefits. Given the predominantly young and educated sample in this study, expanding participant demographics to include varied age groups, educational backgrounds, and socioeconomic levels is crucial. This broader approach would provide richer insights into how body compassion varies across different Iranian contexts and support the development of culturally sensitive interventions. Exploring cultural nuances in how body compassion is interpreted and experienced across various regions of Iran could inform more tailored intervention strategies. Comparative research examining regional, ethnic, or religious influences on body compassion perceptions would further enhance targeted clinical applications.

The clinical implications of the findings underscore the BCS's potential as a valuable instrument in therapeutic settings focused on enhancing positive body image and psychological well‐being among Iranian individuals. Integrating the BCS into clinical assessments can offer therapists deeper insights into clients’ levels of self‐compassion and acceptance of their physical selves, thereby informing personalized treatment plans. The BCS may also aid in identifying individuals at risk for body image‐related issues, facilitating timely intervention and prevention. Incorporating the BCS within psychotherapeutic interventions can enhance treatment outcomes by fostering compassionate self‐views and reducing self‐criticism in clients struggling with body dissatisfaction. The three subscales of the BCS—defusion, common humanity, and acceptance—provide clinicians with a structured framework to address specific dimensions of body compassion during therapy. Through such integration, therapists can promote a more compassionate and accepting attitude toward body image concerns, ultimately supporting holistic well‐being and resilience in the face of societal and cultural pressures related to physical appearance. Future research efforts should focus on broadening the scope and diversity of BCS‐related studies in Iran. Clinical practice can benefit greatly from incorporating this instrument to enhance therapeutic outcomes and support individuals in cultivating a healthier, more compassionate relationship with their bodies.

## 5 Conclusion

This study highlighted the robustness of the BCS, confirming its value as a reliable instrument for both clinical and research purposes within the Iranian context. The BCS's three distinct subscales—defusion, common humanity, and acceptance—provide a comprehensive framework to understand and cultivate self‐compassion among individuals with varying levels of body image concerns. By validating these subscales, this research contributes to the expanding body of evidence supporting the cross‐cultural relevance and applicability of the BCS. Significant correlations were observed between the BCS and other established measures, with particular emphasis on the positive relationship between the acceptance subscale and measures of self‐compassion. These results underscore that embracing one's physical self and acknowledging shared human imperfections are fundamental to fostering compassionate self‐perceptions. Therefore, this study offers empirical support for the psychometric soundness and cultural adaptability of the Persian version of the BCS. The scale's thorough validation, strong psychometric properties, and cultural sensitivity establish it as a valuable tool for advancing research and clinical interventions aimed at enhancing positive body image and self‐compassion in Persian‐speaking populations.

## Author Contributions

**Nahid Hoseininezhad**: conceptualization, methodology, software, data curation, investigation, validation, formal analysis, resources, writing – original draft, writing – review and editing. **Roya Rasuli**: conceptualization, supervision, project administration, investigation, formal analysis. **Abbas Abdollahi**: methodology, software, supervision, conceptualization, investigation, formal analysis. **Kavoos Hassanli**: conceptualization, supervision, data curation, investigation, formal analysis.

## Ethics Statement

This study was conducted in accordance with the ethical standards of the National Research Committee and the 1964 Declaration of Helsinki and its later amendments, or comparable ethical guidelines. Prior to data collection, ethical approval for all study procedures was obtained from Alzahra University (IR/11/04/1401). The approved protocol covered all study procedures, including participant recruitment, informed consent, data collection, and data management. All participants, who were Iranian university students, received a written informed consent form that clearly described the study's aims, procedures, potential risks and benefits, and data confidentiality. Participation was entirely voluntary, and students were informed that they could withdraw at any time without any penalty or consequence. No identifying personal information was collected. All data were anonymized and securely stored in password‐protected electronic files, accessible only to the research team. To ensure cultural appropriateness and minimize potential bias, the research instruments and procedures were carefully adapted and reviewed by experts in psychology and ethics. The rights, safety, and well‐being of all participants were fully protected throughout the research process.

## Consent

All participants provided written informed consent to participate in the study. Their identities were protected by anonymizing their data, replacing names with unique codes.

## Conflicts of Interest

The authors declare no known competing interests or personal relationships that could influence the work reported in this paper.

## Peer Review

The peer review history for this article is available at https://publons.com/publon/10.1002/brb3.70845.

## Data Availability

The data that support the findings of this study are available on request from the corresponding author. The data are not publicly available due to privacy or ethical restrictions.
